# Lannea edulis lowers blood glucose by modulating absorption, utilization, and pancreatic function in diabetic rats

**DOI:** 10.3389/fphar.2025.1618241

**Published:** 2025-08-01

**Authors:** Gilbert Lutangu, Musalwa Muyangwa-Semenova, Rehana Omar, Lubinda Mukololo

**Affiliations:** ^1^Department of Basic Sciences, School of Medicine, University of Lusaka, Lusaka, Zambia; ^2^Department of Physiological Sciences, School of Medicine, University of Zambia, Lusaka, Zambia

**Keywords:** alloxan monohydrate, glucose absorption, hyperglycemia, *Lannea edulis*, pancreas

## Abstract

**Background:**

Diabetes mellitus affects over 537 million people worldwide. However, its management is compounded by factors such as high cost and perceived side effects associated with conventional antidiabetic drugs. This has prompted a rise in alternative therapies, such as medicinal plants. *Lannea edulis* (Sond.) Engl. var. edulis, native to sub-Saharan Africa, has been shown to have both antihyperglycemic and antihyperlipidemic properties, although its mode of action remains unclear. This study investigated the mode of action by which *L. edulis* decreases blood sugar.

**Methodology:**

Aqueous leaf extracts of *L. edulis* obtained by decoction were screened for phytochemicals by qualitative analysis. The effects of the leaf extract (0.25 mg/mL, 0.5 mg/mL and 1.0 mg/mL) on the absorption of glucose in the small intestines using the everted rat jejunum was analysed against controls. The effect of different concentrations of the leaf extracts (1 mg/mL, 2 mg/mL and 2 mg/mL with 1 IU/mL of insulin) on glucose uptake by peripheral tissues was also analysed using isolated rat hemidiaphragms. Lastly, histopathological analyses of the rat pancreas after confirmed alloxan-induced diabetes and subsequent treatment with the leaf extracts at doses of 100 mg/kg and 500 mg/kg for 14 days were carried out against normal rats or diabetic controls treated with 150 mg/kg vitamin C or normal saline.

**Results:**

*L. edulis* extracts contained flavonoids, tannins, phenols and saponins. Treatment with 0.5 mg/mL of the leaf extract significantly decreased the movement of glucose from the mucosal side to the serosa in the everted rat jejunum (p < 0.001) and significantly increased glucose uptake by the hemidiaphragm at 1 mg/mL (p = 0.0029) and 2 mg/mL (p = 0.0479). Dosages of 500 mg/kg of extract improved pancreatic histology in alloxan-induced diabetic rats.

**Conclusion:**

The results show that *L. edulis* significantly reduced intestinal glucose absorption in the rat jejunum model, significantly enhanced glucose uptake in the isolated rat hemidiaphragm, and preserved and promoted regeneration of pancreatic islets and β-cells in diabetic rats. This data supports *L. edulis’s* potential as a complementary therapy in diabetes management.

## Introduction

Diabetes mellitus is a chronic metabolic disease characterized by raised blood glucose levels due to insufficient insulin production by the pancreas, insulin resistance, or both (Bharti et al., 2018). The common characteristics of diabetes mellitus are polydipsia, polyuria, ketonemia, ketonuria, and glycosuria (Thévenod, 2008). There are two main types of diabetes mellitus: type 1 diabetes mellitus, caused by β-cell destruction, usually mediated by immune mechanisms, which leads to absolute insulin deficiency (Müller-Wieland PD med et al., 2019; Forouhi and Wareham, 2019; Kaul et al., 2012; Jiang et al., 2020), and type 2 diabetes mellitus characterized by insulin resistance and defective insulin secretion (Müller-Wieland PD med et al., 2019). While type 1 diabetes mellitus mostly affects a genetically predisposed younger population and requires exogenous insulin administration, type 2 diabetes mellitus typically affects individuals over the age of 40 and is referred to as non-insulin dependent diabetes because low levels of endogenous insulin are still secreted in many patients (Freeman, 2013). In addition to the above, the World Health Organization (WHO) also recognizes gestational diabetes and ketosis-prone type 2 diabetes as other types of diabetes.

The number of diabetes mellitus cases is on the rise, and it is estimated that by the year 2040, about 642 million people will be affected (Kassim et al., 2020). Interestingly, the rise in newly diagnosed type 2 diabetes mellitus cases far exceeds that of type 1 diabetes mellitus (Patterson et al., 2009; Divers et al., 2020; Khan et al., 2020). This has presented a huge economic burden, with global annual expenditures reaching up to 700 billion USD (Usai et al., 2022; Ali et al., 2022). The factors that contribute to the high prevalence of type 2 diabetes mellitus include urbanization, an aging population, decreased levels of physical activity, and an increase in the prevalence of obesity (Wu et al., 2014; Cheema et al., 2014). Indeed, due to these factors, reports indicate that more children as young as 2 years of age, particularly those with a family history of diabetes are now being diagnosed with type 2 diabetes and that this trend correlates with low levels of physical activity and intake of high caloric foods with low nutritional value (Forouhi and Wareham, 2019). In addition, ethnicity, race, and socioeconomic factors also contribute to the rapid rise of diabetes mellitus (Forouhi and Wareham, 2019). Untreated, chronic diabetes mellitus results in renal and retinal pathologies often leading to renal failure and blindness respectively. Furthermore, it significantly increases the risk of hypertension and possible coronary heart disease as well as neuro vascular associated pathologies (D’Souza et al., 2023).

The management of diabetes mellitus involves various strategies such as modification of diet, lifestyle change, intake of oral antihyperglycemic drugs, and/or exogenous administration of insulin depending on the disease subtype (Venkatesh et al., 2010). Despite their effectiveness, these therapies are often associated with adverse side effects, drug resistance, and high costs. These prohibitive factors have led to a rise in use of traditional medicinal plants to treat diabetes mellitus, especially in resource-constrained regions (Nangandu et al., 2022). In sub-Saharan Africa, *Lannea edulis* (Sond.) Engl. var. *edulis*, a member of the Anacardiaceae family, is widely used in ethnomedicine for managing diabetes mellitus (Sekkizhar et al., 2020). Preliminary studies have shown that aqueous leaf extracts of *L. edulis* possess antihyperglycemic and antihyperlipidemic effects in diabetic animal models, likely due to its rich content of flavonoids, saponins, tannins, and phenolic compounds (Banda et al., 2018). However, while its glucose-lowering potential has been observed, the specific mechanism underlying its activity remains poorly understood. Scientific validation of plant-based remedies such as *L. edulis* require mechanistic studies to investigate how they influence key pathways in glucose metabolism. These may include modulation of intestinal glucose absorption, enhancement of peripheral glucose uptake, or preservation of pancreatic β-cell function. Such investigations are essential not only for confirming therapeutic efficacy but also for ensuring safety, optimizing dosing, and guiding future development of plant-derived antidiabetic agents (Shanak et al., 2019). This study therefore sought to investigate the antihyperglycemic mode of action of aqueous leaf extracts of *L. edulis*. Specifically, we aimed to assess the effect of the leaf extracts on intestinal glucose absorption, peripheral glucose utilization, and pancreatic histomorphology using validated *ex-vivo* models and alloxan-induced diabetic Wistar rats. The findings are expected to provide mechanistic insights that support *L. edulis’* traditional use and inform its potential development as a complementary therapeutic option for diabetes management.

## Materials and methods

### Study design

The study adopted a laboratory-based experimental design.

### Study site

The study was conducted in Zambia, a landlocked nation situated in sub-Saharan Africa. Covering a geographical area of 753,612 square kilometers, Zambia is located between latitudes 8° and 18° South and longitudes 22° and 34° East. Phytochemical analyses were conducted at the Department of Chemistry, University of Zambia, while the biochemical analyses were done at the Department of Physiological Sciences, School of Medicine, University of Zambia, Lusaka, Zambia.

### Study population and ethical consideration

The study population comprised Wistar rats obtained from the University of Zambia. This study adopted the guidelines for ethical conduct in the care and use of animals provided by the American Association of Psychologists. Ethical approval was sought from the University of Zambia Biomedical Research Ethics Committee (Ref. No. 2892-2022) and the National Health Research Authority (NHRA-1656/23/10/2024).

### Sample size

The sample size was calculated using R Studio. To determine the effect of the leaf extracts of *L. edulis* on glucose absorption in the everted rat jejunum, the sample size for Kruskal-Wallis for 7 groups was determined. The power level was set at 80%, the significance level at 5%, and a large effect size was assumed. The sample size for 7 groups was determined to be 42. To investigate the effects of the leaf extracts of *L. edulis* on glucose uptake in the isolated hemidiaphragm, the sample size for five groups was determined to be 30. Lastly, to analyze the effects of the leaf extracts of *L. edulis* on the histology of the pancreas, the sample size was determined to be 20 using the same method outlined above.

### Plant collection and identification

*Lannea edulis* whole plant was obtained from Chongwe, 47 km east of Lusaka, with the help of a field assistant. The plant was then taken to the Department of Biological Sciences at the University of Zambia for identification and authentication by a qualified botanist. A voucher specimen with accession number 22523 was deposited at the herbarium situated in the School of Natural Sciences, Department of Biological Sciences at The University of Zambia.

### Preparation of aqueous leaf extracts

The leaves of *L. edulis* were washed with running water to eliminate surface contaminants and air-dried in the shade for 7 days. This was followed by blending the leaves to homogeneity. The blended leaves were then placed in hot water and allowed to sit for 30 min. The water was then filtered using MN615 150 mm filter paper. The filtrate was evaporated to dryness using a hot water bath at 40°C to obtain a sticky brownish residue. The residue was weighed, stored in air and water-proof containers, and then kept between 4°C and 8°C. A fresh preparation was made from this stock whenever required.

### Percentage yield determination

The percentage yield was determined as follows:

$$Percentage\;yield=\frac{Mass\;of\;extract\;\left(g\right)}{Mass\;of\;ground\;leaves\;\left(g\right)\;}X100\%$$

### Phytochemical screening

The presence of various secondary metabolites in the leaf extracts of *L. edulis* was assessed using different standard procedures (Shaikh and Patil, 2020; Altemimi et al., 2017; Abuzaid et al., 2020; Nortjie et al., 2022; Pandey et al., 2014; Patil and Deshmukh, 2016). We assessed the presence of phenols, flavonoids, saponins, glycosides, steroids, terpenoids, alkaloids, carbohydrates, and amino acids.

### Measurement of glucose uptake in an isolated rat hemidiaphragm

Glucose uptake by the rat diaphragm was examined according to standard protocol (Mohamed et al., 2013). Rats weighing 150–200 g were sacrificed by cervical dislocation and dissected to remove the diaphragm. Each diaphragm was rinsed in ice-cold Tyrode’s solution to preserve the tissue, and then cut into two equal hemidiaphragms, which were gently blotted and weighed. The composition of Tyrode’s solution is as follows: NaCl (134 mM), KCl (2.68 mM), CaCl_2_ (1.80 mM), MgCl_2_ (1.05 mM), NaH_2_PO_4_ (417 µM), NaHCO_3_ (11.9 mM), and glucose (5.56 mM). This solution is a balanced salt solution that is isotonic with interstitial fluid and is used in experiments to mimic normal physiological conditions (Barreto-Chang and Dolmetsch, 2009; Gilani et al., 2001). Each hemidiaphragm was placed in a test tube containing 2 mL of Tyrode’s solution and test sample with constant aeration by bubbling air throughout the experiment. Since Tyrodes’ reagent contains glucose, well-preserved tissues are expected to take up glucose from the solution. The tissues were constantly shaken at 37°C and incubated for 90 min.

Triplicate samples of each medium were taken for glucose estimation before the placement of the tissues and at the end of the experiment. The hemidiaphragms were randomly placed in one of the five groups (n = 5) as shown in Table 1. Equal numbers of left and right hemidiaphragms were used for controls and test samples, respectively and dosages of the leaf extract used in the study were established after preliminary tests were conducted using a similar study for reference (Mohamed et al., 2013). The results obtained were expressed as the amount of glucose utilized by the hemidiaphragms per gram of tissue during the 90-min incubation period. The glucose concentration was measured using an Accu-Chek glucose meter. The dosages used are commonly employed in studies involving the isolated hemidiaphragm (Mohamed et al., 2013).

**TABLE 1:** Experimental groups.

Glucose uptake by the isolated rat hemi-diaphragm			Glucose absorption by the everted rat jejunum			Effect of leaf extracts on pancreatic histology of rats		
Group (n = 5)	Treatment	Concentration	Group (n = 6)	Treatment	Concentration	Group (n = 6)	Treatment	Concentration
1	Insulin	1 IU/mL	1	Control	Tyrodes’ Solution	1	Control	Tyrodes’ Solution
2	*Lannea edulis*	1 mg/mL	2	*Lannea edulis*	0.25 mg/mL	2	*Lannea edulis*	0.25 mg/mL
3	*Lannea edulis*	2 mg/mL	3	*Lannea edulis*	0.5 mg/mL	3	*Lannea edulis*	0.5 mg/mL
4	Insulin + *Lannea edulis*	2 mg/mL + 1 IU/mL	4	*Lannea edulis*	1.0 mg/mL	4	*Lannea edulis*	1.0 mg/mL
5	Control	Tyrodes’ Solution	5	Metformin	0.75 mg/mL	5	Metformin	0.75 mg/mL
			6	Metformin	1.0 mg/mL			
			7	Metformin	3.0 mg/mL			

### Measurement of glucose absorption in the everted rat jejunum

Healthy experimental rats that weighed between 150 and 200 g were fasted for 16 h before the experiment. They were then sacrificed by a cervical dislocation, and dissected to remove the small intestine. The jejunum segments were excised and placed in ice-cold Tyrode’s solution, a physiological solution used to maintain tissue viability. Then, the segments were rinsed with Tyrode’s reagent, and everted onto a glass rod.

The segments were then cut into 5 cm pieces (n = 6), rinsed, and placed on filter paper to dry before being weighed. Each piece of the intestine was tied tightly around one end with a thread, while the other end only required a loose knot (Mohamed et al., 2013). Thereafter, 2 mL of Tyrode’s solution was injected into each sample using a slipping blunt needle, and the knot was tightened.

The jejunum samples were randomly placed in seven groups (n = 6), and then incubated in 30 mL Tyrode’s solutions as shown in Table 1. Metformin was used because different studies have shown that it inhibits intestinal glucose transport at varying concentrations (Horakova et al., 2019). The preparations were constantly aerated by bubbling air and kept at 37°C throughout the experiment. At the end of the 90-min incubation period, the glucose concentration of the inside sacs and the incubation media was determined using the Accu-chek glucose meter. Results were expressed as milligrams of glucose absorbed per gram of tissue (Mohamed et al., 2013).

### Histological morphology of the pancreas of experimental rats

Alloxan monohydrate (Sigma Aldrich) was used to cause pharmacological diabetes by intravenously injecting 60 mg/kg of Alloxan monohydrate dissolved in 0.9 percent w/v cold normal saline to overnight-fasted rats (12 h). The rats were then kept on a 10% glucose solution for 24 h to prevent hypoglycemia. After 72 h of injection, fasting blood glucose level was measured, and animals that developed more than 200 mg/dL glucose levels were considered diabetic. The rats were randomly allocated to five groups (n = 6) and subjected to different oral treatments for 14 days, as shown in Table 1. Toxicity studies have been conducted on the leaf extracts of the *L. edulis*, and the LD_50_ was found to be greater than 6,000 mg/kg, and according to Hodge and Sterner, falls in the practically nontoxic range (Banda et al., 2018). This was referenced in this study to make sure the dosages of *L. edulis* used are safe and effective. At the end of the experimental period, rats from each group were surgically operated on under diethyl ether (1.9%) anesthesia (at 80 μL per litre of volume of a container). The pancreatic tissues were dissected out, and placed in 10% neutral buffered formalin and fixed in PBS containing 10% formalin. The tissues were then washed in running tap water, dehydrated in descending grades of isopropanol, and finally cleared in xylene. The tissues were thereafter embedded in molten paraffin wax, cut into transverse 5 μm sections of the mid-organ level, and stained with Hematoxylin-Eosin (Venkatesh et al., 2010; Mohamed et al., 2013; Al-Qudah et al., 2016; Atangwho et al., 2012). Histopathological changes in the pancreatic tissues were observed under a light microscope at a magnification of 400X. The number of islets and the number of β-cells in each islet were counted by three pathologists.

### Quantification of pancreatic islet area

Pancreatic islet area was quantified using ImageJ (NIH). The pixel-to-micron scale was calibrated based on the microscope camera sensor and optical system, yielding a scale of 0.0875 µm/pixel. Using the freehand selection tool, individual islets of Langerhans were outlined manually, and their cross-sectional areas were measured in µm^2^. A minimum of four islets per treatment were analyzed, and mean islet areas were compared across experimental groups using One-Way ANOVA.

### Data analysis

The data was initially collected in MS Excel 2019 and exported to Stata version 17 for analysis. Data normality was determined using box plots, and it was found to be not normally distributed. This distribution was due to the small sample size that the study used. Given that data were not normally distributed, the median and interquartile range were used to summarize it. The Kruskal-Wallis non-parametric test and One-Way ANOVA were used to determine whether there were statistically significant differences between two or more intervention groups for glucose uptake and glucose absorption. Tukey’s HSD test and Dunn’s test with Bonferroni correction were used for *post hoc* testing. Data are presented as mean ± SD unless otherwise specified; graphical representations show mean ± SEM. The results were considered statistically significant if *p* value was less than 0.05.

## Results

### Plant collection and leaf extract preparation

**FIGURE 1:**
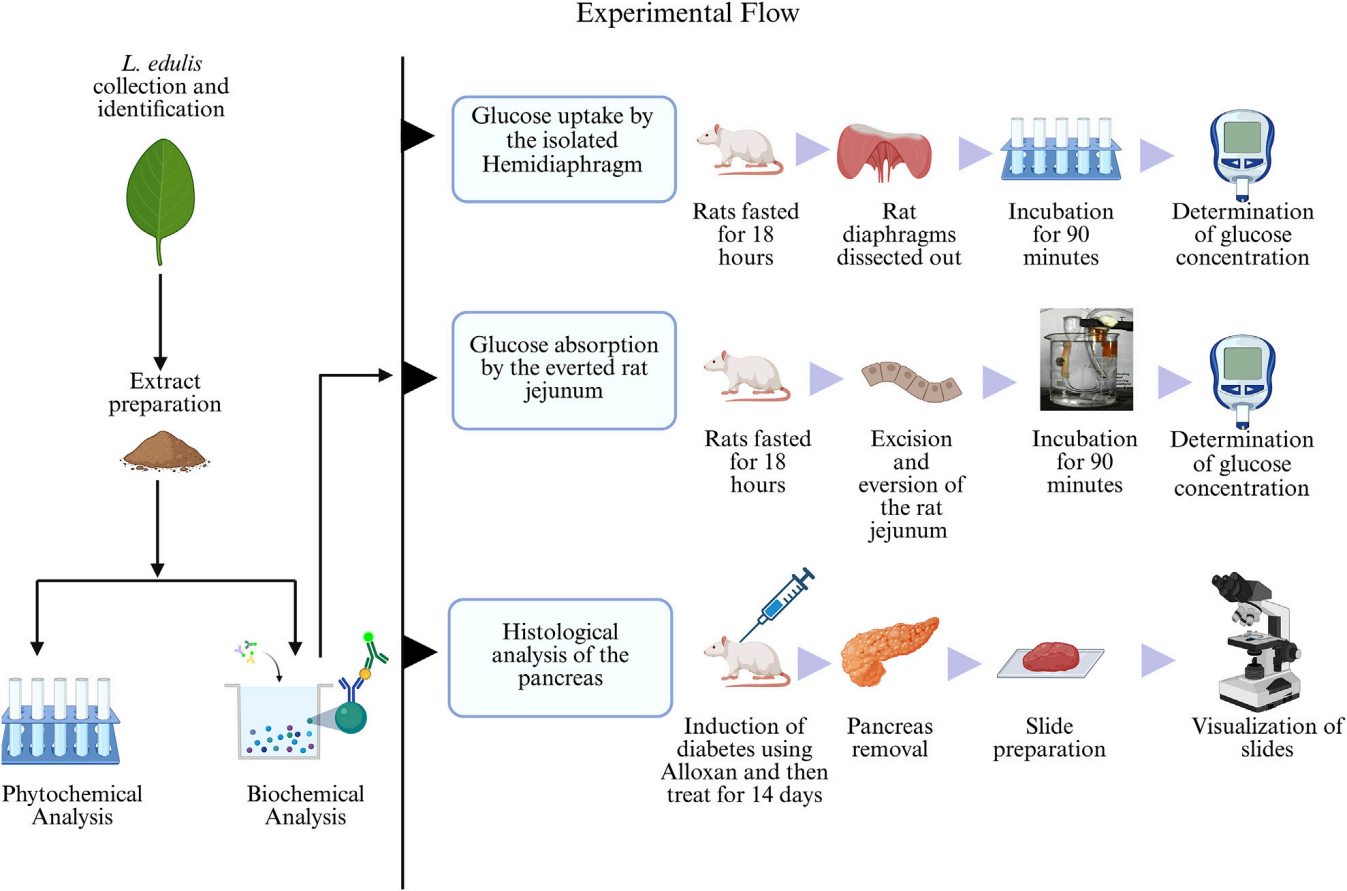
Experimental flow of the study. The leaf extracts of *Lannea edulis* were first screened for phytochemicals, followed by biochemical analyses to determine glucose absorption and pancreatic tissue morphology. Glucose uptake by the isolated rat hemidiaphragm was determined by fasting rats for 18 h, dissection, and incubation of their diaphragms in Tyrode’s solution and leaf extracts before glucose concentration measurement using a glucometer. Glucose absorption by the everted rat jejunum was determined by fasting rats for 18 h followed by dissection and preparation of jejunum sacs. The jejunum sacs were then incubated in Tyrode’s solution and leaf extracts before measuring glucose concentration with a glucometer. Histological morphology of rat pancreases was determined by induction of diabetes using Alloxan monohydrate followed by treatment with leaf extracts for 14 days. After dissections, pancreatic tissue slides were prepared and visualized under a light microscope. (Created with BioRender.com).

*Lannea edulis* whole plant was collected from Chongwe, Zambia, with the help of a field assistant. The plant was then taken to the University of Zambia, Department of Biological Sciences, for professional identification and authentication after which it was assigned the unique accession number 22523. Following leaf extract preparation, the percentage yield of 13.1% was obtained; this is less than the amount reported previously (Banda et al., 2018). The *L. edulis* aqueous leaf extract was then used for phytochemical and biochemical analyses, as illustrated in Figure 1.

### Qualitative phytochemical screening

Several studies have reported the presence of phytochemicals in the leaf extracts of *L. edulis* (Banda et al., 2018; Kumar et al., 2023; Queiroz et al., 2003). To confirm this, the leaf extracts of *L. edulis* were screened for the presence of alkaloids, saponins, steroids, terpenoids, glycosides, flavonoids, phenols, tannins, carbohydrates, amino acids, and proteins. Our data shows that phytochemicals were present in varying amounts (Table 2).

**TABLE 2:** Phytochemical screening of *Lannea edulis* leaf extract.

SN	Phytochemicals	Inference	Color intensity
1	Phenol/Tannins	++	Moderate color intensity
2	Flavonoids	++	Moderate color intensity
3	Saponins	++	Moderate color intensity
4	Glycosides	+	Low color intensity
5	Steroids	+	Low color intensity
6	Terpenoids	+	Low color intensity
7	Alkaloids	+	Low color intensity
8	Amino Acids	+	Low color intensity
9	Carbohydrates	+	Positive
10	Reducing sugars	+	Low color intensity

Key: (++) = present-*moderate amount*; (+) = present-*low amount*; (−) = absent.

### Effects of leaf extracts of *Lannea edulis* on the uptake of glucose in the isolated rat hemidiaphragm

**FIGURE 2:**
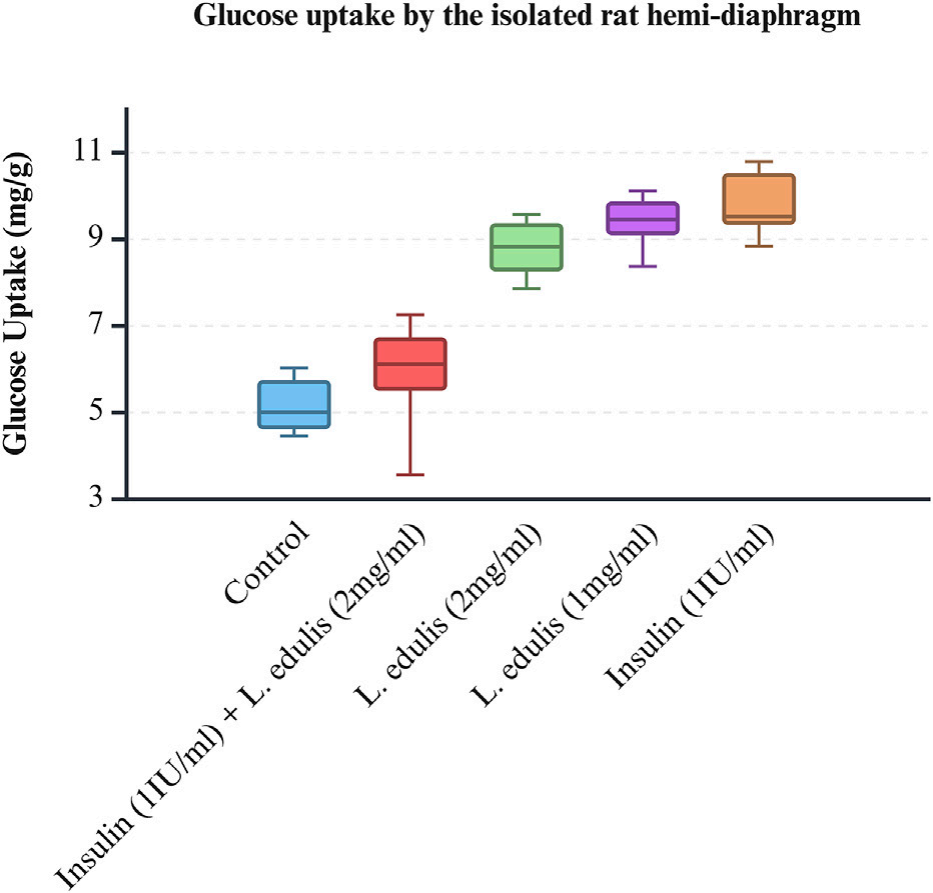
Glucose uptake by the isolated rat hemi-diaphragm model. A box plot of glucose uptake by the isolated rat hemidiaphragm. Data was plotted from the median glucose absorption by the isolated hemidiaphragm initially incubated in Tyrode’s solution and varying concentrations of *Lannea edulis* leaf extract.

The hemidiaphragm model is an *ex-vivo* system commonly used to study muscle glucose metabolism and evaluate anti-diabetic agents (Buse et al., 1976; Sabu and Subburaju, 2002; Koski and Max, 1974; Bati et al., 2017; Fernandes et al., 2007). To study the effects of *L. edulis* on glucose uptake in the isolated rat hemidiaphragm, five groups were established as shown in Materials and Methods. Our data, as illustrated in Table 3, indicates that the median glucose uptake in milligrams in the isolated rat hemidiaphragm model was highest in the insulin 1 IU/mL group [9.51 (9.33–10.75)] and lowest in the control group [4.98 (4.61–5.83)]. This data was further visualized using box plots (Figure 2). Our results show that the data were not normally distributed, indicating that the uptake of glucose in the isolated rat hemidiaphragm model varied across treatment groups. Given that our data was not normally distributed, we performed a Kruskal-Walli’s test to check if glucose uptake across groups was significantly different (Table 4). Our results show that there was a significant difference between the intervention groups and the negative control in the uptake of glucose in the hemidiaphragm model (*p* = 0.001). We then carried out a Dunn’s test to determine which of the interventions had a statistically significant effect. A *post hoc* test using Dunn’s test with Bonferroni correction, as shown in Table 5, showed that glucose uptake in the hemidiaphragm model was significantly higher in the *L. edulis* 1 mg/mL (p = 0.0029), *L. edulis* 2 mg/mL (*p* = 0.0479), and insulin 1 IU/mL (*p* = 0.0007) groups compared to the control. Additionally, the *L. edulis* 1 mg/mL group (*p* = 0.0266) and the insulin 1 IU/mL group (*p* = 0.0082) had significantly higher glucose uptake than the insulin 1 IU/mL + *L. edulis* 2 mg/mL group. This data suggests chemical antagonism between the leaf extracts of *L. edulis* and insulin.

**TABLE 3:** Glucose uptake in the isolated rat hemidiaphragm model.

Intervention	Median (interquartile range) (mg/g of tissue)
Insulin 1 IU/mL	9.51 (9.33–10.75)
*Lannea edulis* 1 mg/mL	9.44 (9.06–9.88)
*Lannea edulis* 2 mg/mL	8.81 (8.14–9.43)
Insulin 1 IU/mL + *Lannea edulis* 2 mg/mL	6.09 (5.35–6.85)
Control 0 mg/mL *Lannea edulis*	4.98 (4.61–5.83)

**TABLE 4:** Effects of *Lannea edulis* leaf extracts on glucose uptake in the isolated rat hemi-diaphragm model.

Intervention	Observation	Rank sum	P value
Control	6	29	0.0001
Insulin 1 IU/mL	6	145	
Insulin 1 IU/mL + *Lannea edulis* 2 mg/mL	6	49	
*Lannea edulis* 1 mg/mL	6	134	
*Lannea edulis* 2 mg/mL	6	108	

**TABLE 5:** Glucose uptake in the isolated rat hemidiaphragm model.

Comparison	Z Statistics	P Value
Control 0 mg/mL - *Lannea edulis* 1 mg/mL	−3.44	**0.0029**
Control 0 mg/mL - *Lannea edulis* 2 mg/mL	−2.59	**0.0479**
Control 0 mg/mL - Insulin 1 IU/mL	−3.8	**0.0007**
Control 0 mg/mL - Insulin 1 IU/mL + *Lannea edulis* 2 mg/mL	−0.65	1.000
*Lannea edulis* 1 mg/mL - *Lannea edulis* 2 mg/mL	0.85	1.000
*Lannea edulis* 1 mg/mL - Insulin 1 IU/mL	−0.36	1.000
*Lannea edulis* 1 mg/mL - Insulin 1 IU/mL + *Lannea edulis* 2 mg/mL	2.79	**0.0266**
*Lannea edulis* 2 mg/mL - Insulin 1 IU/mL	−1.21	1.000
*Lannea edulis* 2 mg/mL - Insulin 1 IU/mL + *Lannea edulis* 2 mg/mL	1.93	0.2651
Insulin 1 IU/mL - Insulin 1 IU/mL + *Lannea edulis* 2 mg/mL	3.15	**0.0082**

Bold values represent statistically significant results.

### Effect of leaf extracts of *Lannea edulis* on glucose absorption in the everted rat jejunum model

**FIGURE 3:**
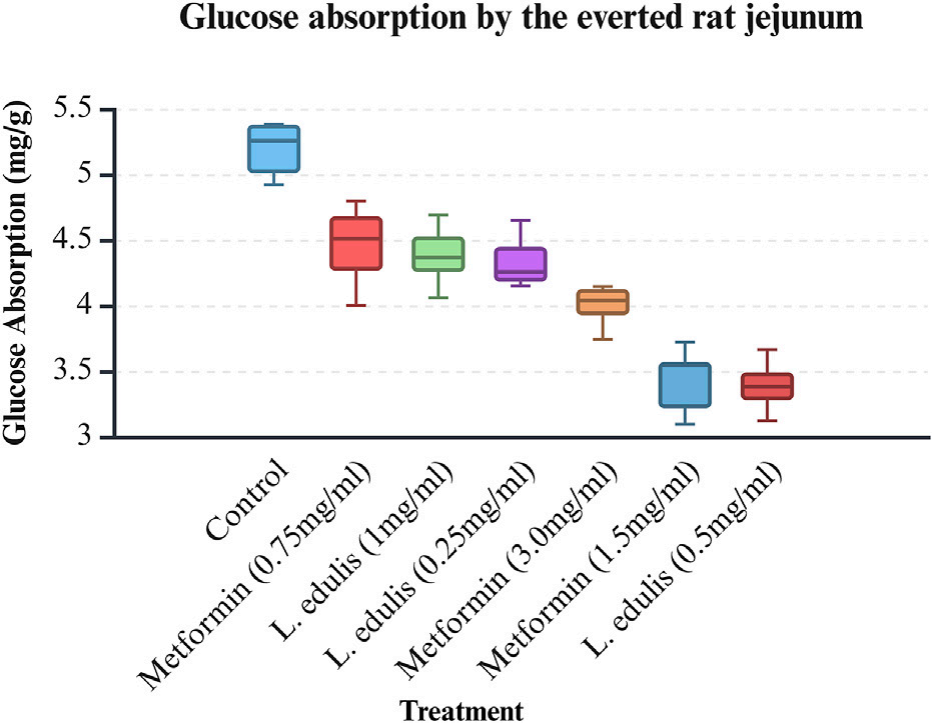
Glucose absorption by the everted rat jejunum model. A box plot of glucose uptake by the everted rat jejunum. Data was plotted from the median glucose absorption by the everted jejunum initially incubated in Tyrode’s solution and varying concentrations of *Lannea edulis* leaf extract.

The everted rat jejunum is an *ex-vivo* experimental model that mimics the physiological environment of the small intestine and is often used to study early-stage absorption (Kellett and Helliwell, 2000; Mace et al., 2007; Morgan et al., 2007; Alam et al., 2012; Emoto et al., 2000; Wilcock and Bailey, 1991). To evaluate the effects of *L. edulis* on the absorption of glucose in the everted rat jejunum, seven groups were formed, as shown in Materials and Methods. Our data, as shown in Tables 6, 8 indicates that the median absorption of glucose in the everted rat jejunum model was highest in the negative control group [5.26 (4.95–5.37)] and lowest in the *L. edulis* group (0.5 mg/mL) [3.38 (3.27–3.54)]. This data was further visualized using a box plot (Figure 3). Our data shows that there were variations in absorption of glucose by different treatment groups, suggesting that some interventions might have inhibited glucose absorption in the everted rat jejunum. The data was skewed, an indication the data was not normally distributed; this suggests that the extent of inhibition was not dependent on the concentration of the interventions. Since our data was not normally distributed, we performed a Kruskal-Walli’s test to confirm the differences in glucose absorption observed in Figure 3. As shown in Table 7, our results indicate that there was a significant difference in glucose absorption in the inverted rat jejunum model between the interventions and the negative control (*p* = 0.001). This data suggests that some interventions lowered the amount of glucose absorbed. Additionally, we carried out a Dunn’s test to determine which of the interventions had a statistically significant effect. A *post hoc* test using Dunn’s test with Bonferroni correction as shown in Table 8 indicated that glucose absorption in the everted rat jejunum was significantly inhibited by *L. edulis* 0.5 mg/mL (*p* < 0.001), metformin (1.5 mg/mL) (*p* < 0.001) and metformin (3.0 mg/mL) (*p* = 0.0043). Further, *L. edulis* (0.5 mg/mL) significantly inhibited glucose absorption than *L. edulis* (0.25 mg/mL) and (1 mg/mL) groups (*p* = 0.0273 and *p* = 0.0136). *Lannea edulis* (0.5 mg/mL) significantly lowered glucose absorption than the metformin group (0.75 mg/mL) (*p* = 0.007). The absorption of glucose was significantly higher in the metformin (0.75 mg/mL) group than in the metformin (1.5 mg/mL) group (*p* = 0.0098). Collectively, this data shows that the leaf extracts of *L. edulis* significantly decreased glucose absorption in the everted rat jejunum at a concentration of 0.5 mg/mL.

**TABLE 6:** Absorption of glucose in the everted rat jejunum model (mg/g of tissue).

Interventions	Median (interquartile range) (mg)
Control	5.26 (4.95–5.37)
Metformin (0.75 mg/mL)	4.51 (4.20–4.70)
*Lannea edulis* (1 mg/mL)	4.37 (4.25–4.53)
*Lannea edulis* (0.25 mg/mL)	4.26 (4.17–4.47)
Metformin (3.0 mg/mL)	4.04 (3.93–4.11)
Metformin (1.5 mg/mL)	3.55 (3.22–3.56)
*Lannea edulis* (0.5 mg/mL)	3.38 (3.27–3.54)

**TABLE 7:** Effect of leaf extracts of *Lannea edulis* on the absorption of glucose in the everted rat jejunum model.

Interventions	Observation	Rank sum	P value
Control	7	322	**0.0001**
Metformin (0.75 mg/mL)	7	232	
*Lannea edulis* (1 mg/mL)	7	222	
*Lannea edulis* (0.25 mg/mL)	7	211	
Metformin (3.0 mg/mL)	7	133	
Metformin (1.5 mg/mL)	7	55	
*Lannea edulis* (0.5 mg/mL)	7	50	

Bold values represent statistically significant results.

**TABLE 8:** Glucose absorption in the everted rat jejunum model.

Comparisons	Z Statistics	P Value
Control - *Lannea edulis* (0.25 mg/mL)	2.08	0.3977
Control - *Lannea edulis* (0.5 mg/mL)	5.09	**<0.001**
Control - *Lannea edulis* (1 mg/mL)	1.87	0.6449
Control - Metformin (0.75 mg/mL)	1.68	0.9691
Control - Metformin (1.5 mg/mL)	4.99	**<0.001**
Control - Metformin (3.0 mg/mL)	3.53	**0.0043**
*Lannea edulis* (0.25 mg/mL) - *Lannea edulis* (0.5 mg/mL)	3.01	**0.0273**
*Lannea edulis* (0.25 mg/mL) - *Lannea edulis* (1 mg/mL)	−0.2	1.000
*Lannea edulis* (0.25 mg/mL) - Metformin (0.75 mg/mL)	−0.39	1.000
*Lannea edulis* (0.25 mg/mL) - Metformin (1.5 mg/mL)	2.92	**0.037**
*Lannea edulis* (0.25 mg/mL) - Metformin (3.0 mg/mL)	1.46	1.000
*Lannea edulis* (0.5 mg/mL) - *Lannea edulis* (1 mg/mL)	**−3.22**	**0.0136**
*Lannea edulis* (0.5 mg/mL) - Metformin (0.75 mg/mL)	−3.4	**0.007**
*Lannea edulis* (0.5 mg/mL) - Metformin (1.5 mg/mL)	−0.09	1.000
*Lannea edulis* (0.5 mg/mL) - Metformin (3.0 mg/mL)	−1.55	1.000
*Lannea edulis* (1 mg/mL) - Metformin (0.75 mg/mL)	−0.19	1.000
*Lannea edulis* (1 mg/mL) - Metformin (1.5 mg/mL)	3.12	**0.0188**
*Lannea edulis* (1 mg/mL) - Metformin (3.0 mg/mL)	1.66	1.000
Metformin (0.75 mg/mL) - Metformin (1.5 mg/mL)	3.31	**0.0098**
Metformin (0.75 mg/mL) - Metformin (3 mg/mL)	1.85	0.6727
Metformin (1.5 mg/mL) - Metformin (3 mg/mL)	−1.46	1.000

Bold values represent statistically significant results.

### Effect of *Lannea edulis* leaf extracts on pancreatic tissue of diabetic rats

**FIGURE 4:**
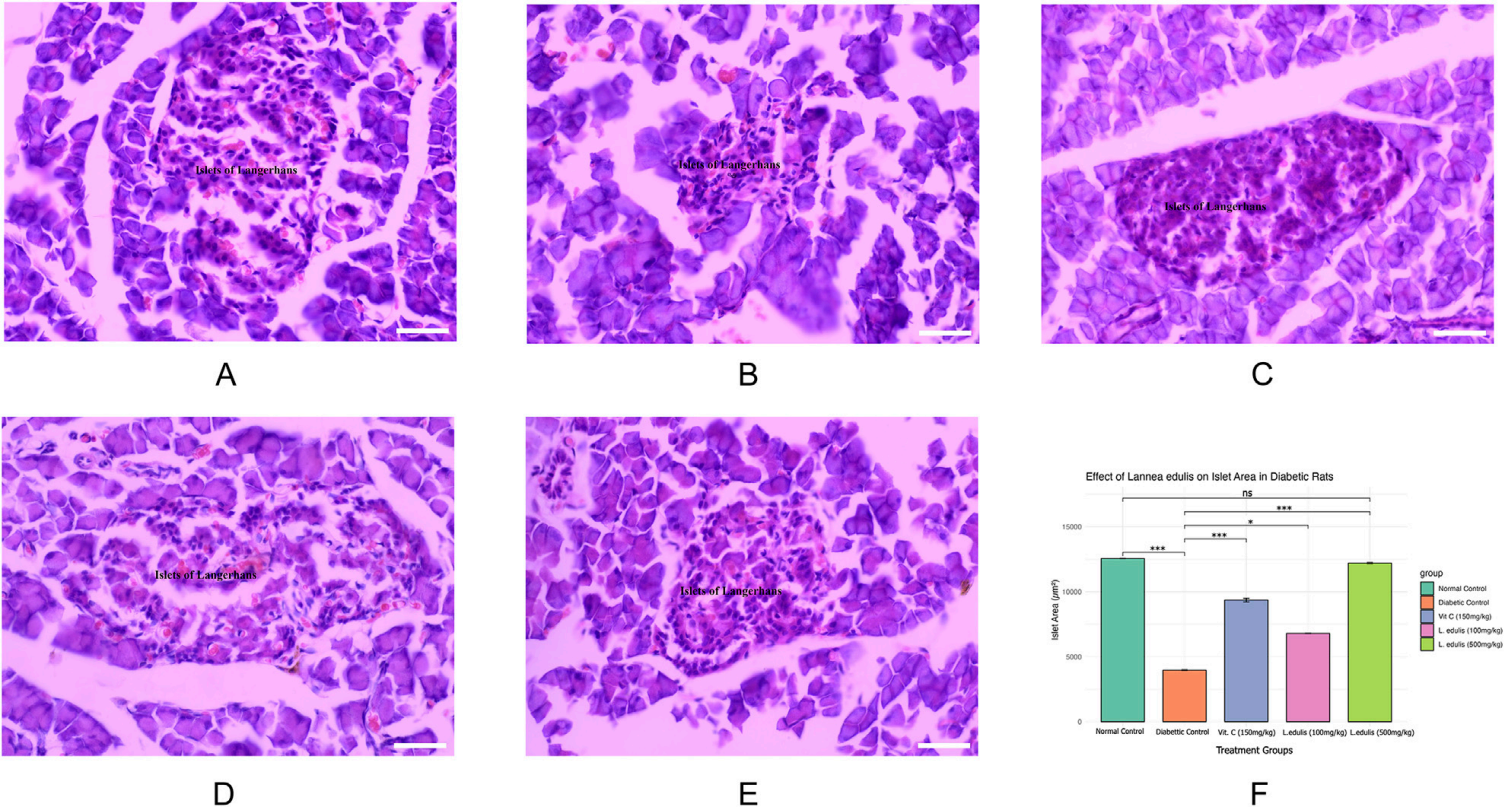
Effect of *Lannea edulis* leaf extracts on diabetic rat pancreas. **(A–E)** High power-view of pancreatic tissue sections from the normal control group treated with normal saline, diabetic group treated with normal saline, diabetic group treated with vitamin C (150 mg/kg), diabetic group treated *Lannea edulis* (500 mg/kg), and diabetic group treated with *Lannea edulis* (100 mg/kg), respectively. Tissues were stained with hematoxylin and eosin and slides visualized under a light microscope at X400. **(F)** Bar graph showing mean pancreatic islet area (µm^2^) ± SEM across treatment groups: Normal Control, Diabetic Control, Vitamin C (150 mg/kg), *L. edulis* (100 mg/kg), and *L. edulis* (500 mg/kg). Islet area was quantified using ImageJ following H&E staining. Statistical analysis was performed using one-way ANOVA followed by Tukey’s HSD test. Scale Bar: 30 μM *p < 0.05, **p < 0.01, ***p < 0.001, ns = not significant.

The baseline fasting blood glucose levels were within the normal range, measuring between 3.3 mmol/L and 4.2 mmol/L before diabetes induction. Alloxan, used to induce diabetes in the current experiment, selectively destroys pancreatic β-cells (Lenzen, 2008; Malaisse et al., 1982). Diabetes was confirmed by post induction blood glucose levels of more than 200 mg/dL. Further, the experimental diabetic rats exhibited classical signs of diabetes mellitus, including polydipsia, polyuria, observable lethargy, and progressive weight loss. To analyze the effect of *L. edulis* leaf extracts on the histological morphology of β-cells in the pancreas of rats, we prepared pancreatic sections from the five groups as described in Materials and Methods, following induction of diabetes and administration with the extracts. Our data shows that the sections obtained from the normal control group treated with normal saline exhibited normal pancreatic histology and normal islet structure (Figure 4A). Furthermore, our data showed that the pancreatic sections from the alloxan-induced diabetic group treated with normal saline showed very few islets, with a notable reduction in size (Figure 4B). The high-power view photomicrographs of the pancreatic sections from the alloxan-induced diabetic group treated with vitamin C (150 mg/kg) showed islets close to normal size with signs of β-cell regeneration (Figure 4C). The high-power view photomicrographs of the pancreatic sections from the alloxan-induced diabetic group treated with *L. edulis* (500 mg/kg) showed normal-sized islets with regeneration of β-cells (Figure 4D). The photomicrographs of sections obtained from the alloxan-induced diabetic group treated with a lower dose of *L. edulis* (100 mg/kg) showed reduced islet size and reduced number of β-cells (Figure 4E). To confirm if there were significant differences in the size of islets among the five treatment groups, we quantified the islet areas as earlier described and then performed a One-Way ANOVA and Tukey HSD test (Figure 4E). Our data revealed significant differences in islet area across the experimental groups (One-Way ANOVA, *p* < 0.0001). As expected, diabetic control rats exhibited a marked reduction in islet area (mean: 3,968.83 ± 61.18 µm^2^) compared to normal controls (mean: 12,559.92 ± 36.48 µm^2^), confirming alloxan-induced β-cell destruction (*p* < 0.001, Tukey HSD test). Treatment with vitamin C (150 mg/kg) significantly increased islet area to 9,358.81 ± 281.25 µm^2^ (*p* < 0.001 vs. diabetic control), indicating partial regenerative potential. Notably, administration of *L. edulis* extract at 100 mg/kg modestly improved islet area (6,794.73 ± 26.41 µm^2^), while the 500 mg/kg dose almost completely restored islet size (12,202.05 ± 87.13 µm^2^), with no statistically significant difference from the normal control group (*p* > 0.05). These findings demonstrate a dose-dependent β-cell protective and regenerative effect of *L. edulis* extract in diabetic rats. To confirm if there were significant differences in the number of islets and the number of β-cell among the five treatment groups, we performed the Kruskal-Walli’s test. The results comparing the number of β-cells indicated a statistically significant difference between the groups (χ^2^ (6) = 27.204, *p* = 0.0001) (Table 9). This suggests that at least one of the treatment groups had a significantly different number of β-cells from the others. For the number of islets, the analysis also revealed a statistically significant difference among these groups (χ^2^ (6) = 25.227; *p* = 0.0001) (Table 9). These results indicated that at least one of the treatment groups had a significantly different distribution (or median, assuming similar distribution shapes) of the number of islets compared to the others. We performed Dunn’s test to identify specific differences in β-cell count among the various treatment groups following a significant Kruskal-Walli’s test (Table 10). The group treated with Vitamin C (150 mg/kg) showed significantly higher β-cell count compared to *L. edulis* (100 mg/kg) (*p* = 0.0058). The diabetic control group treated with normal saline (1 mL) had significantly lower β-cell ranks than the groups treated with Vitamin C (150 mg/kg) (*p* = 0.0001) and *L. edulis* (500 mg/kg) (*p* = 0.0091). This is consistent with other studies that have reported the regenerative effects of Vitamin C on the pancreas (Gui et al., 2023). The normal control group treated with normal saline (1 mL) showed significantly higher β-cell ranks compared to *L. edulis* (500 mg/kg) (*p* = 0.0140), *L. edulis* (100 mg/kg) (*p* = 0.0004), and especially diabetic control group treated with normal saline (1 mL) (*p* = 0.00001). No significant differences were observed between the groups treated with Vitamin C (150 mg/kg) and *L. edulis* (500 mg/kg) (*p* = 0.0893). These findings suggest that *L. edulis* at the dosage of 500 mg/kg could have regenerative effects on the β-cells of the pancreas. Additionally, Dunn’s test was performed to identify specific differences in islets among the various treatment groups following a significant Kruskal-Walli’s test (Table 11). The results show that there was no significant difference in islet ranks between the groups treated with Vitamin C (150 mg/kg) and *L. edulis* (500 mg/kg) (*p* = 0.0812). The diabetic control group treated with normal saline had significantly lower islet ranks compared to the groups treated with *L. edulis* (500 mg/kg) (*p* = 0.0253) and *L. edulis* (100 mg/kg) (*p* = 0.0366). These unadjusted results show that the groups treated with *L. edulis* generally lead to higher islet ranks compared to the diabetic control group, suggesting possible regenerative and augmentation effects of the leaf extracts of *L. edulis* on the islets. Together, this data suggests that at higher doses, the leaf extracts of *L. edulis* have an antioxidant and regenerative effect on the pancreas.

**TABLE 9:** Kruskal-Wallis test for number of islets and β-cells by treatment group.

Variable	Groups	N (total)	χ^2^ (H)	df	p
Islets	5	30	25.227	4	0.0001
β-cells	5	30	27.204	4	0.0001

**TABLE 10:** Dunn’s Pairwise comparisons for number of β-cells by group.

Group	Diabetic control: Vitamin C (150 mg/kg)	*Lannea edulis* (500 mg/kg)	*Lannea edulis* (100 mg/kg)	Diabetic control: Normal saline (1 mL)
*Lannea edulis* (500 mg/kg)	1.345191			
*p*	0.0893			
*Lannea edulis* (100 mg/kg)	2.526335	1.18114		
*p*	0.0058	0.1188		
Diabetic control: Normal saline (1 mL)	3.707479	2.362287	1.181144	
*p*	0.0001	0.0091	0.11188	
Normal control: Normal saline (1 mL)	−0.853048	−2.198239	−3.379383	−4.560527
*p*	0.1968	0.0140	0.0004	0.0000

**TABLE 11:** Dunn’s pairwise comparisons for number of islets by group.

Group	Diabetic control: Vitamin C (150 mg/kg)	*Lannea edulis* (500 mg/kg)	*Lannea edulis* (100 mg/kg)	Diabetic control: Normal saline (1 mL)
*Lannea edulis* (500 mg/kg)	1.396897			
*p*	0.0812			
*Lannea edulis* (100 mg/kg)	1.561238	0.164341		
*p*	0.0592	0.4347		
Diabetic control: Normal saline (1 mL)	3.352552	1.955655	1.791315	
*p*	0.0004	0.0253	0.0366	
Normal control: Normal saline (1 mL)	−1.380463	−2.777359	−2.941700	−4.733015
*p*	0.0837	0.0027	0.0016	0.0000

## Discussion

*Lannea edulis* is widely used as an alternative medicine in the management of diabetes in sub-Saharan Africa (Deutschlnder et al., 2009). In this study, we investigated the potential antihyperglycemic mode of its aqueous leaf extract using *ex-vivo* and histological analyses. Our findings demonstrated that *L. edulis* exerts antidiabetic effects through a combination of pathways, consistent with previous reports about phytochemical-rich plant extracts (Modak et al., 2007; Al-Ishaq et al., 2019).

The phytochemical screening of the aqueous leaf extract revealed the presence of phenols, flavonoids, tannins, and saponins, confirming earlier reports on *L. edulis* and other medicinal plants used for diabetes management (Queiroz et al., 2003; Chandran et al., 2017; Abdel-Sattar et al., 2017; Rodríguez-García et al., 2019; Maniyar et al., 2012; Zehad et al., 2017). These compounds have been shown to improve insulin sensitivity, enhance glucose uptake, and protect β-cells through antioxidant activity (Modak et al., 2007; Al-Ishaq et al., 2019; Zheng et al., 2020). Alkaloids, for example, have been reported to have antihyperglycemic activity due to their ability to inhibit the enzyme α-glucosidase (Pan et al., 2003; van de Laar, 2008). Furthermore, alkaloids exert a potent stimulating effect on the basal glucose uptake rate (Sharma and Gupta, 2017). Berberine stimulated insulin secretion in a dose-dependent manner in rats’ pancreatic islets, via a pathway involving hepatic nuclear factor 4α (Wang et al., 2008; Liu et al., 2005). Terpenes from the stem of *Paeonia suffruticosa* were able to increase glucose uptake and enhance glycogen synthesis by activating AMP-activated protein kinase (AMPK) in HepG-2 cells, while Flavonoids exerted antidiabetic effects via different mechanisms (Ha et al., 2009). Tannins have been shown to possess antidiabetic and antioxidant properties (Hii and Howell, 1984; Hii and Howell, 1985). Interestingly, the results of this study showed that phenols were among the major class of phytochemicals identified and this data is in line with published reports (Maroyi and Semenya, 2019). It is important to note that phenolic compounds are known to inhibit α-amylase activity and exhibit antidiabetic activity by increasing glucose uptake in adipocytes (Suryanarayana et al., 2004). It is therefore plausible that phenols in *L. edulis* play a major role in glucose uptake; however, individual assessment of the mechanism/s of action of these major phytochemicals is required. These phytochemicals in *L. edulis* may work alone or in synergy to exert their antidiabetic activity.

One notable finding was the extract’s capacity to inhibit glucose absorption in the rat intestinal model, which points to a possible interaction with intestinal glucose transport mechanisms. Previous reports have shown that phytochemicals such as flavonoids, a type of polyphenol, and tannins may block sodium-glucose co-transporter 1 (SGLT1) and the glucose transporter type 2 (GLUT2) (Song et al., 2002; Cermak et al., 2004). Glucose absorption through the intestinal epithelium mainly involves SGLT1, which transports glucose against its concentration gradient (Tofighi et al., 2017). The diffusive component of glucose absorption is mediated by the recruitment of GLUT 2 to the apical membrane (Wright et al., 2011). Our data suggests that *L. edulis* may inhibit the activity of SGLT1 and the translocation of GLUT 2 to the apical membrane, thereby reducing glucose entry into circulation post-ingestion. This mechanism is shared by other dietary phytochemicals, including plant polysaccharides and polyphenols (Cui and Li, 2025). It has also been reported that the activity of SGLT 1 induces a cytoskeletal rearrangement that loosens and widens the intercellular spaces. Given the potential effect of *L. edulis* leaf extract on SGLT 1, there may be limited cytoskeletal rearrangement, thereby inhibiting the transport of glucose across tight junctions (Gromova et al., 2021; Kellett et al., 2008). In our model, the 0.5 mg/mL concentration of the extract appeared more effective than the higher dose (1 mg/mL), hinting at the kind of non-linear interactions often observed with extract constituents as reported earlier (Kan et al., 2017). In line with these data, reports have shown that high doses of certain phytochemicals such as polyphenols can have adverse effects including increased production of reactive oxygen species (Chen et al., 2014), reduced cell viability (Brondani et al., 2020) as well as liver and kidney toxicity (Granat et al., 2018). Given that certain compounds such as flavonoids exhibit increased pancreatic β-cell survival and function via a reduction in oxidative stress pathways, downregulation of pro-apoptotic genes and increased translocation of pro-inflammatory cytokines from β-cells (Ghorbani et al., 2019), it can be speculated that high doses may exert the reduced efficacy seen in our data.

We observed that *L. edulis* leaf extract enhanced glucose uptake in isolated rat hemi-diaphragm tissue (a model of skeletal muscle glucose utilisation). At 1 mg/mL, glucose uptake increased to levels comparable to insulin-treated tissues, suggesting insulin-like properties. This effect is possibly mediated via the translocation of glucose transporter type 4 (GLUT4) to the plasma membrane, either through PI3K/Akt or AMPK signaling pathways, as has been observed with other flavonoid-rich extracts such as those from *Morus alba* and *Camellia sinensis* (Zang et al., 2006; Kang et al., 2022). This mode of action may offer an advantage in insulin-resistant conditions, where insulin signaling is impaired but alternative pathways like AMPK remain responsive.

Histological analysis further showed that *L. edulis* leaf extract preserved pancreatic islet structure and cellular integrity in alloxan-induced diabetic rats. The quantification data demonstrates the potential of *L. edulis* leaf extract in protecting and regenerating pancreatic islets in alloxan-induced diabetic rats, supporting its value as a phytotherapeutic candidate. The restoration of islet architecture observed with the extract treatment underscores the critical role of antioxidative mechanisms in mitigating β-cell damage caused by oxidative stress, a central feature in alloxan-induced diabetes (Lenzen, 2008; Szkudelski, 2001). Plant-derived antioxidants have been widely recognized for their ability to preserve β-cell mass and function by modulating oxidative stress and inflammatory pathways within pancreatic tissue (Robertson, 2004; Deng et al., 2021), and the dose-dependent improvement seen with *L. edulis* aligns with this established paradigm. Our data also highlights the potential for *L. edulis* in contributing to endogenous insulin production, which is pivotal for glycemic control in diabetes management (Poitout and Robertson, 2002). Moreover, the observed regenerative effects are consistent with evidence that phytochemicals can promote β-cell proliferation while reducing apoptosis under diabetic conditions (Kumar et al., 2023; Rolo and Palmeira, 2006). This is consistent with reports that flavonoids like quercetin and chrysin promote β-cell regeneration and reduce oxidative damage in pancreatic tissue (El-Sayyad et al., 2011; Srinivasan et al., 2005). Not surprisingly, the propensity of various plant extracts for wound healing and tissue regeneration is attributed to their antioxidant and anti-inflammatory effects (Iqbal et al., 2024). Indeed, a study showed that *L. edulis* extracts contain radical-scavenging bioactive compounds that may reduce oxidative damage (Queiroz et al., 2003). The improved histoarchitecture seen in our extract-treated groups, particularly at 500 mg/kg, implies protective and possibly regenerative effects, likely mediated by antioxidant phytochemicals.

Collectively, our data suggests that *L. edulis* effects its antidiabetic property in three ways: (i) inhibition of intestinal glucose absorption via modulation of SGLT1 and GLUT2 activity; (ii) stimulation of peripheral glucose uptake likely through insulin-independent pathways such as AMPK activation; and (iii) restoration of pancreatic islet structure, possibly via antioxidant-mediated cytoprotection. This data provides evidence for the multifaceted role of *L. edulis* in hyperglycemic control and supports its potential application in the management of diabetes mellitus. While conventional therapies remain central, growing interest in β-cell replacement and regenerative strategies, including islet xenotransplantation (Matsumoto and Yamamoto, 2023) and patient-derived pancreatic models (Gu et al., 2024), underscores the importance of diversifying therapeutic approaches. In this context, validating traditional medicines such as *L. edulis*, provides a complementary pathway with both cultural and biomedical value.

## Conclusion

We evaluated the antidiabetic mode of action of the aqueous leaf extracts of *L. edulis*, commonly used in sub-Saharan Africa for managing diabetes. Phytochemical screening revealed the presence of key secondary metabolites, including flavonoids, tannins, phenols, and saponins, which are known for their glucose-lowering and antioxidant properties. Using alloxan-induced diabetic rats and *ex-vivo* models that included the everted rat jejunum and isolated hemi-diaphragm, we demonstrated that the extract reduces intestinal glucose absorption, enhances peripheral glucose uptake, and promotes pancreatic β-cell preservation and regeneration. Our data provides mechanistic insights into the traditional use of *L. edulis*, supports its ethnomedical relevance, and identifies it as a promising candidate for further development as a plant-based complementary therapy for diabetes. The clearly defined model systems and phytochemical profiles used in this study enhance the reproducibility of results and provide a framework for future pharmacological and clinical investigations.

## Study limitations

We would like to acknowledge that although crude phytochemical analysis was conducted, the inclusion of liquid chromatography mass spectrometry (LC-MS) to accurately identify and quantify the individual compounds present in the aqueous extract of *L. edulis* as opposed to the broad selected groups of phytochemicals would have been more insightful. Furthermore, the activity of these compounds individually or their combined potential synergistic activity would have enabled us to better understand their mechanism of action on glucose absorption as well as their effect/s on prescribed anti-diabetic drugs. The present study was conducted over a few weeks and thus does not allow us to speculate on the long-term efficacy or indeed toxicological effects of *L. edulis*. To this end, a longitudinal research design is warranted before this can be translated into clinical trials. Future studies assessing whether *L. edulis* increases β-cell secretion of the pancreas or whether it interacts with glucose transporters thus improving insulin sensitivity is needed in order to appreciate the effects of this extract at the molecular level. While this study uses a model that simulates type 1 diabetes mellitus, it would be interesting to stratify the study by the type of diabetes to test the effects of the herbal extract in childhood and adult on-set diabetes especially given that some aspects of the types of diabetes mellitus have overlapping etiologies.

## Recommendations

We recommend the following:

1. Further studies should be conducted to isolate, characterize, and identify the bioactive compounds responsible for the observed effects of *L. edulis*.
2. Cellular mechanisms by which these leaf extracts affect effect their antihyperglycemic activity should be elucidated; these studies may include evaluating the effects of the isolated active ingredients on β-cell function, glucose transporters, and on the activity of the main enzymes involved in glucose metabolism.
3. Detailed toxicological evaluations should be carried out to establish the safety profile of the extracts.
4. The long-term efficacy and potential synergistic effects with existing antidiabetic drugs should be investigated.
5. Validation of the clinical effectiveness of our results using patient-derived models over an extended observation period with regular patient check-ups.

## Data Availability

The original contributions presented in the study are included in the article/Supplementary Material, further inquiries can be directed to the corresponding author.
