# Supplementary material for: Lannea edulis lowers blood glucose by modulating absorption, utilization, and pancreatic function in diabetic rats
Source: Front Pharmacol. 2025 Aug 1;16:1618241. doi: 10.3389/fphar.2025.1618241 (PMC12354538; doi:10.3389/fphar.2025.1618241)
Supplement: Supplementary file 1 [file Table1.docx]

**S1: Effect of *Lannea eduli*s on Islet Area in Diabetic Rats**

| **Treatment Group** | **Islet Area (µM^2^)** | | | | |  |
| --- | --- | --- | --- | --- | --- | --- |
|  | 1 | 2 | 3 | 4 | **X̄** | **SD (**σ**)** |
| Normal Control: Normal saline (1ml) | 12515.32 | 12545.22 | 12585.70 | 12593.43 | 12559.92 | 36.48 |
| Diabetic Control: Normal saline (1ml) | 4038.80 | 3940.81 | 3996.17 | 3899.54 | 3968.83 | 61.18 |
| Diabetic: Vitamin C (150mg/kg) | 9773.69 | 9291.04 | 9198.45 | 9172.06 | 9358.81 | 281.25 |
| Diabetic: *Lannea edulis* (100 mg/kg) | 6769.53 | 6819.71 | 6774.38 | 6815.28 | 6794.73 | 26.41 |
| Diabetic: *Lannea edulis* (500 mg/kg) | 12109.29 | 12282.77 | 12269.63 | 12146.50 | 12202.05 | 87.13 |
